# Impacts of COVID-19 pandemic on kidney biopsy research, practice, and diagnoses: A cross-sectional audit

**DOI:** 10.1097/MD.0000000000045597

**Published:** 2025-11-07

**Authors:** Rachel M. Brennan, Benjamin Strimaitis, Yilin Tian, Benjamin Adams, Robert J. Ellis, Shaun Chandler, Dharmenaan Palamuthusingam, Sharad Ratanjee, Dwarakanathan Ranganathan, Kirsten Hepburn, Eoin D. O’Sullivan, George John, Brian Doucet, Helen G. Healy, Andrew Kassianos, Monica Suet Ying Ng

**Affiliations:** a Kidney Health Service, Royal Brisbane and Women’s Hospital, Brisbane, Queensland, Australia; b Conjoint Internal Medicine Laboratory, Chemical Pathology, Pathology Queensland, Brisbane, Queensland, Australia; c Faculty of Health, Medicine and Behavioural Science, University of Queensland, Brisbane, Queensland, Australia; d Griffith School of Medicine and Dentistry, Griffith University, Gold Coast, Queensland, Australia; e Cancer Program, QIMR Berghofer Medical Research Institute, Brisbane, Queensland, Australia; f Institute of Molecular Bioscience, University of Queensland, Brisbane, Queensland, Australia; g Infection and Inflammation Program, QIMR Berghofer, Brisbane, Queensland, Australia.

**Keywords:** complications, coronavirus, COVID-19 pandemic, glomerular disease, kidney biopsy, research

## Abstract

The impact of the coronavirus disease 2019 (COVID-19) pandemic extended beyond direct infection-associated complications to wide-reaching impacts on health system including workflow disruption, enhanced telehealth utilization, labor force changes, and access to procedures. Whilst it is clear that COVID-19 can affect histopathological findings on kidney biopsy, the impact of COVID-19 pandemic on non-COVID-19 kidney disease research remains unclear. This study reviewed kidney biopsy research activity (i.e., patient consent and bio-sample collection), kidney biopsy practice and histopathological findings over the COVID-19 pandemic in an Australian metropolitan health service network of 4 hospitals between 2018 and 2023. All kidney biopsies performed at the Metro North Kidney Health Service between 2018 and 2023 were divided into pre-pandemic (2018–2019), pandemic (2020–2021) and post-pandemic (2022–2023) eras. Demographic data, consent rates, bio-sample collection rates, and procedural complications were retrospectively compared between the 3 eras. Two hundred twenty-nine kidney biopsies were performed in 2018 to 2019, 223 in 2020 to2021 and 213 in 2022 to 2023. Participant consent for research reduced from 70% to 63% between pre-pandemic to pandemic eras but quickly recovered in the post-pandemic era (68%). Bio-sample collection decreased (pre-pandemic: 50%, pandemic: 47%, post-pandemic: 38%) and did not recover in the post-pandemic era indicating the long tail effect on research activities. Although there were changes in service provision (e.g., delay in elective procedures, lockdowns), these measures were not associated with changes in biopsy number, setting, and department over the course of the pandemic. Kidney disease biomarker research activities decreased during the COVID-19 pandemic as demonstrated by reductions in biomarker study consent and sample collection. Strategies to maintain non-pandemic research need to be built into pandemic preparedness plans to preserve the momentum of discoveries which improve clinical outcomes for non-pandemic diseases; discoveries which may well end up being repurposed for pandemic-related conditions (e.g., remdesivir from Ebola research).

## 1. Introduction

The impact of the coronavirus disease 2019 (COVID-19) pandemic extended beyond direct infection-associated complications. The COVID-19 pandemic had wide-reaching impacts on the health care system including clinical workflow disruption to reduce severe acute respiratory syndrome coronavirus 2 (SARS-CoV-2) transmission, enhanced telehealth utilization, labor force changes and access to procedures such as kidney biopsy.^[1]^ Moreover, public health measures such as lockdowns reduced general population access to nonurgent services and research staff access to clinical spaces for non-COVID-19 research.^[2]^ Rapid escalation and redirection of resources to develop COVID-19 vaccines and therapies reduced resources available for non-pandemic-related research activities.^[3]^

On January 29, 2020, Queensland was the first Australian state to declare a public health emergency.^[4]^ Measures imposed intermittently over 2020 to 2021 included closure of state borders, limitations of patron numbers for public gatherings, restrictions to aged care and hospital visitors, elective procedure delays and school closures. Queensland’s COVID-19 vaccination program started in March 2021, achieving an 86.6% double dose vaccination rate by end of the year. Vaccination facilitated the easing of restrictions in early 2022. COVID-19 was declared to be no longer a Communicable Disease Incident of National Significance on October 20, 2023. The Queensland COVID-19 mortality rate was 33.9 per 1,00,000, lower than the national mortality rates per 1,00,000 of 77 in Australia, 341 in United States of America, 255 in France, 135 in Canada and 58 in Japan.^[5,6]^

Whilst it is clear that COVID-19 can affect histopathological findings on kidney biopsy,^[7]^ the impact of the COVID-19 pandemic on non-COVID-19 kidney disease research remains unclear. The Metro North Kidney Health Service is a network of hospitals with longstanding kidney disease biomarker discovery studies that span the pre-pandemic (2018–2019), pandemic (2020–2021), and post-pandemic (2022–2023) eras, enabling assessment of kidney disease research engagement over the course of the COVID-19 pandemic. We evaluated kidney biopsy practice, histopathological findings, research recruitment, and sample collection from 2018 to 2023. All kidney biopsies performed at the Metro North Kidney Health Service between 2018 and 2023 were divided into pre-pandemic (2018–2019), pandemic (2020–2021), and post-pandemic (2022–2023) eras and retrospectively analyzed.

## 2. Materials and methods

The Metro North Human Research Ethics Committee (HREC) waived ethics review for the project as it was considered a quality improvement audit (EX/2023/MNHA/97565). All kidney biopsies performed at the Metro North Kidney Health Service between January 1, 2018 and December 31, 2023 were audited. Biopsies were divided into pre-pandemic (2018–2019), pandemic (2020–2021), and post-pandemic (2022–2023) eras. The pre-pandemic era was characterized by an absence of Australian COVID-19 cases, the pandemic era was defined as the period with the most stringent public health suppression measures in place, and the post-pandemic era was characterized by the revocation of many suppressive public health measures (e.g., border restrictions and quarantine) and transition of government policy from “no community transmission” to “living with COVID-19.”^[8,9]^ Demographic, laboratory, procedural, histopathology, complication, and research engagement data was extracted from the medical chart by 5 investigators (BS, YT, BA, AK, MSYN).

The Metro North Kidney Health Service has longstanding biomarker studies (identification of molecular pathways induced by proteinuria in the human kidney proximal tubule compartment [HREC/2002/QRBW/011], Biomarker in Kidney Disease study [BioKiD, HREC/2022/QRBW/81341]) with a research workflow. Potential participants are approached regarding interest in contributing to ongoing research studies at time of biopsy by clinical or research staff. Consented participants provide relevant bio-samples (e.g., urine, blood, excess kidney tissue from kidney biopsy after diagnostic analysis) which are collected by research staff. These samples then enter relevant research study pathways per HREC-approved study protocol. In this audit, surrogate indicators of kidney disease research engagement were participant consent for biomarker studies and excess kidney tissue received from kidney biopsies.

Data were compared between the 3 eras (2018–2019, 2020–2021, 2022–2023). Continuous data were presented as either mean ± standard deviation if normally distributed, or median [interquartile range] if not normally distributed, and compared using a standard or Kruskal–Wallis analysis of variance, respectively. Categorical data were reported as count (%) and compared using a Chi-square test. Univariable and multivariable logistic regression was used to assess the 2 ancillary outcomes: bleeding; and obtaining a consent for biospecimen research. Adjustment was made for confounders, ascertained using directed acyclic graphs. Tukey’s test was used to account for multiple comparisons. All analysis was performed using Stata 14 (StataCorp, USA).

To contextualize changes in kidney biopsy-related research output over time, a search of studies indexed in the Pubmed database which cited kidney biopsies was undertaken, with results presented descriptively in the Supplementary Material, Supplemental Digital Content, https://links.lww.com/MD/Q5156.

## 3. Results

There were 229 kidney biopsies performed in 2018 to 2019, 223 in 2020 to 2021 and 213 in 2022 to 2023 (Table 1). The mean age at time of kidney biopsy was similar across the 3 phases of the COVID-19 pandemic. There were more inpatient than outpatient biopsies across all eras. Native kidney biopsies made up over 80% of the kidney biopsies in all eras. There were more patients biopsied on dialysis during COVID-19 although this did not reach statistical significance (*P* = .07). The leading indication for native kidney biopsy in the pre-pandemic era was impaired kidney function (n = 50, 26%), proteinuria during the pandemic era (n = 65, 36%) and hemoproteinuria (n = 51, 27%) in the post-pandemic era. Creatinine rise was the most prevalent indication for transplant biopsies across all eras.

**Table 1:** Demographic data.

Characteristic	2018–2019 (n = 229)	2020–2021 (n = 223)	2022–2023 (n = 213)	*P*-value
Age (yr), mean ± SD	52 ± 18	51 ± 16	51 ± 18	.77
Sex, n (%)				
M	118 (52)	114 (51)	107 (50)	.93
F	111 (48)	109 (49)	106 (50)	
Proceduralist, n (%)				
Nephrology	175 (76)	177 (79)	160 (75)	.67
IR	54 (24)	46 (21)	53 (25)	
Location				
Inpatient	130 (57)	123 (55)	108 (51)	.37
Outpatient	99 (43)	100 (45)	105 (49)	
Kidney, n (%)				
Native	196 (86)	183 (82)	189 (89)	.17
Transplant	33 (14)	40 (18)	24 (11)	
Needle gauge, n				
14	0	1	0	.28
16	144	119	118	
18	75	88	75	
20	2	0	0	
Not recorded	8	15	20	
SBP (mm Hg), mean ± SD	130 ± 13	131 ± 14	131 ± 14	.67
Dialysis, n (%)	46 (20)	50 (22)	30 (14)	.07
Biopsy on aspirin, n(%)	40 (17)	45 (20)	40 (19)	.70
Biopsy indication (native)	196 (86)	183 (82)	189 (89)	<.001^*^
Haematuria†	7 (4)	5 (3)	5 (3)	
Proteinuria†	53 (27)	65 (36)	52 (28)	
Nephrotic syndrome†	13 (7)	19 (10)	15 (8)	
Haemoproteinuria†	28 (14)	31 (17)	51 (27)	
Impaired kidney function†	50 (26)	33 (18)	18 (10)	
Monoclonal gammopathy†	3 (2)	0 (0)	6 (3)	
Lupus nephritis†	15 (8)	12 (7)	27 (14)	
Systemic vasculitis†	6 (8)	15 (8)	15 (8)	
Other†	0 (0)	2 (1)	1 (1)	
Biopsy indication (transplant)	33 (14)	40 (18)	24 (11)	.67
Creatinine rise‡	25 (76)	32 (80)	20 (83)	
Proteinuria‡	2 (6)	3 (8)	1 (5)	
Haemoproteinuria‡	0 (0)	4 (10)	0 (0)	
BK viraemia‡	6 (18)	3 (8)	2 (10)	
Investigations				
Hb, g/L, mean ± SD	115 ± 23	119 ± 24	118 ± 23	.16
Plt, ×10^9^/L, mean ± SD	264 ± 108	273 ± 98	266 ± 99	.62
Urea, mmol/L, mean ± SD	12.7 ± 8.0	12.9 ± 14.2	12.6 ± 8.2	.95
Creatinine, μmol/L, mean ± SD	213 ± 191	188 ± 160	197 ± 152	.28
uPCR, g/mol creat, median [IQR]	154 [57–375]	180 [39–536]	229 [72–499]	.12
uACR, g/mol creat, median [IQR]	112 [27–260]	121 [12–415]	129 [32–310]	.09

IR = interventional radiology, SBP = systolic blood pressure, SD = standard deviation. *P < .001.

† Percentages expressed as percentage of native kidney biopsies over period.

‡ Percentages expressed as percentage of transplant kidney biopsies over period.

In the pre-pandemic era, 70% (n = 160) of patients undergoing a kidney biopsy consented for biomarker research. The consent rate trended downwards during the pandemic era to 63% (n = 141) but did not reach statistical significance (Table 2). Post-pandemic, the proportion of people undergoing kidney biopsy consenting to biomarker research returned to pre-pandemic levels at 68% (n = 145). The proportion of biopsy samples sent for research declined precipitously from 50% (n = 115) in the pre-pandemic era, to 47% (n = 104) in the pandemic era, to 38% (n = 81) in the post-pandemic era (*P* = .03).

**Table 2:** Likelihood of research consent being signed.

Characteristic	Research consent signed		OR (95% CI)	*P*-value
	No (n = 219)	Yes (n = 446)		
Year				
2018–2019 (229)	69 (30)	160 (70)	1	
2020–2021 (223)	82 (37)	141 (63)	0.74 (0.50–1.09)	.14
2022–2023 (213)	68 (32)	145 (68)	0.92 (0.61–1.38)	.68
Location				
Inpatient (361)	132 (37)	229 (63)	1	
Outpatient (304)	87 (29)	217 (71)	1.27 (0.94–1.73)	.12
Proceduralist				
Nephrology (512)	80 (26)	432 (84)	1	
IR (153)	139 (91)	14 (9)	0.02 (0.01–0.03)	<.001*

Data reported as count (row %). Odds ratio (OR) and 95% confidence intervals (95% CI) estimated using multivariable logistic regression, with both location and proceduralist adjusted for as confounders.

CI =confidence interval, IR = interventional radiology, OR = odds ratio.

* *P* < .001.

Of all biopsies performed, there were more glomerular disease diagnoses in the post-pandemic era (Table S1, Supplemental Digital Content, https://links.lww.com/MD/Q5156). In contrast, diabetic nephropathy made up 16% (n = 32) pre-pandemic, 17% (n = 32) pandemic, and 11% (n = 20) post-pandemic native kidney biopsies. The ratio of primary glomerular disease:diabetic nephropathy diagnoses was higher in the post-pandemic era compared to the pandemic era (*P* = .042). Tubulointerstitial disease, vascular disease, and deposition disease made up similar proportions of native kidney biopsy diagnoses across the pre-pandemic, pandemic, and post-pandemic eras. Of transplant biopsies, rejection comprised of 24% (n = 8) pre-pandemic biopsies, 18% (n = 7) pandemic biopsies, 29% (n = 7) post-pandemic biopsies. BK nephropathy was more common in the pre-pandemic era at 9% (n = 3), with no cases during the pandemic era and 4% (n = 1) in the post-pandemic era. Across all eras, females were at higher risk of a bleeding complication compared to males. Bleeding risk was highest in the 25 to 34 year old age group with an odds ratio of 2.61 (1.26–5.41; Tables S2 and S3, Supplemental Digital Content, https://links.lww.com/MD/Q5156).

The number of publications citing kidney biopsies dropped from 700 to 750 per 100,000 between 2015 and 2019 to a nadir of ~500 per 1,00,000 between 2022 and 2023. This increased to ~650 per 1,00,000 in 2024 (Fig. S1, Supplemental Digital Content, https://links.lww.com/MD/Q5156).

## 4. Discussion

This is the first review of kidney disease research practices over the course of the COVID-19 pandemic in a jurisdiction with comparatively fewer COVID-19 cases. Kidney disease biomarker research activities decreased during the COVID-19 pandemic as demonstrated by reductions in biomarker study consent and sample collection. Although there were changes in service provision (e.g., delay in elective procedures, lockdowns), these measures were not associated with changes in biopsy number, setting, and department over the course of the pandemic.

The number of participants consented for kidney disease biomarkers studies trended down during the COVID-19 pandemic. At the Metro North Kidney Health Service, people undergoing a kidney biopsy are routinely approached regarding participation in kidney disease biomarkers studies by research and clinical staff. Research samples are collected by research staff. Reductions in research consent and sample collection during the pandemic era could be attributed to restrictions in research staff access to clinical spaces during the pandemic, staff redeployment for COVID-19 purposes and shift in public perceptions around science.^[10]^ Recovery of research consent rates in the post-pandemic era is consistent with staff being reinstated in pre-pandemic positions. The observed fall in excess kidney sample collection over the COVID-19 pandemic was likely due to reduction in available tissue and consent rates. The observation that bio-sample collection rates are yet to recover at the end of the pandemic despite increases in consent rates demonstrated the long tail to the pandemic and its impacts on research workflows. Possible contributors to this long tail effect could include changes to research personnel through redeployment of joint research/clinical staff, potential losses of funding opportunities secondary to reduced research output or altered funding priorities, and reduced medical research student enrollment during the pandemic in the context of limiting hospital access to essential personnel only and a significant shift to remote learning.^[11,12]^

Our findings reflect a wider trend observed in the literature with a reduction in publications citing kidney biopsies over the pandemic era (Fig. S1, Supplemental Digital Content, https://links.lww.com/MD/Q5156). This change occurred in a dynamic landscape where research priorities shifted dramatically towards addressing the global pandemic. This included national and international policies to expedite COVID-19 research ethics/governance reviews, and many journals peer review/publication processes were expedited/prioritized for COVID-19 research.^[13]^ Within the first 6 months of the pandemic there were more than 1000 registered clinical trials related to COVID-19 research.^[13,14]^ The shifting prioritization may explain the reduction in kidney biopsy-related research to some degree.

The incidence of glomerular disease diagnoses were elevated, but not significantly, over the course of the pandemic. These results conflict with the findings from a multicentre cohort of 240 native kidney biopsies of people with COVID-19 which demonstrated increased risks of collapsing glomerulopathy, myoglobin cast nephropathy, proliferative glomerulonephritis with monoclonal immunoglobulin G deposits; and reduced risks of diabetic mellitus, immunoglobulin A nephropathy and arteriosclerosis.^[15]^ Another series of 14 native kidney biopsies in patients with COVID-19, n = 10 (71%) had glomerular pathology (n = 7 of whom had intercurrent tubular pathology) whereas n = 3 (29%) had isolated tubular pathology, indicating a broad range of findings associated with COVID-19 infection.^[16]^ The Kidney Histopathology After COVID-19 and SARS-CoV-2 Vaccination study (NCT05043168) found similar incidence rates of necrotizing glomerulonephritis, thrombotic microangiopathy (TMA) and primary podocytopathies between 2019 and 2021.^[17]^ In that study, only 27 biopsies were completed after COVID-19 infection and 15 biopsies were completed after SARS-CoV vaccination out of 10,206 biopsies between 2019 and 2021, suggesting that COVID-19-related cases comprised only a very small proportion of kidney biopsies, similar to our locale. The higher ratio of primary glomerular disease:diabetic nephropathy diagnoses during the pandemic era observed in our center likely represented reduced appetite to biopsy people with suspected diabetic nephropathy during the pandemic. This was in the broader context of limited resource availability and rationalization of lower-yield care during the COVID-19 pandemic,^[18]^ which may have led to nephrologists being less likely to biopsy a patient with diabetes where there was a high pretest probability of diabetic nephropathy, and the biopsy would have been less likely to alter management. A survey of 182 Australasian nephrologists undertaken before the start of the COVID-19 pandemic (2019–2020) indicated there was already a lower tendency to biopsy people with vs without diabetes who had active urinary sediment (69% vs 83%) or nephrotic range proteinuria (21% vs 93%), supporting this assertion.^[19]^

Limitations of our analysis include use of retrospective data which increases risk of confounding bias however, demographic features were similar across the 3 pandemic eras. Further studies are urgently needed to assess and address impacts on non-pandemic research to increase preparedness for future pandemics. Strategies to maintain non-pandemic research need to be built into pandemic preparedness plans to preserve the momentum of discoveries which improve clinical outcomes for non-pandemic diseases; discoveries which may well end up being repurposed for pandemic-related conditions (e.g., remdesivir from Ebola research). This study also highlights the importance of having robust research infrastructure in place with effective failsafe mechanisms to protect against disruptions to workflows caused by external influences.

## Author contributions

**Conceptualization:** Monica Suet Ying Ng.

**Data curation:** Rachel M. Brennan, Benjamin Strimaitis, Yilin Tian, Benjamin Adams, Robert J. Ellis, Andrew Kassianos, Monica Suet Ying Ng.

**Formal analysis:** Rachel M. Brennan, Robert J. Ellis.

**Investigation:** Rachel M. Brennan, Shaun Chandler, Dharmenaan Palamuthusingam, Sharad Ratanjee, Dwarakanathan Ranganathan, Kirsten Hepburn, Eoin D. O'Sullivan, George John, Brian Doucet, Helen G. Healy.

**Methodology:** Rachel M. Brennan, Robert J. Ellis, Monica Suet Ying Ng.

**Supervision:** Monica Suet Ying Ng.

**Writing – original draft:** Rachel M. Brennan.

**Writing – review & editing:** Benjamin Strimaitis, Yilin Tian, Benjamin Adams, Robert J. Ellis, Shaun Chandler, Dharmenaan Palamuthusingam, Sharad Ratanjee, Dwarakanathan Ranganathan, Kirsten Hepburn, Eoin D. O'Sullivan, George John, Brian Doucet, Helen G. Healy, Andrew Kassianos, Monica Suet Ying Ng.

## References

[1] Cutler DM. Health system change in the wake of COVID-19. JAMA Health Forum. 2023;4:e234355. 37856097 10.1001/jamahealthforum.2023.4355

[2] Feng YR Li I Kristoffersen I Armstrong BK Preen DB. Effect of COVID-19 lockdowns on quality-of-life and health services access by socio-economic status in Australia. Health Promot Int. 2024;39:daae096. 39166485 10.1093/heapro/daae096 PMC11336672

[3] Yanow SK Good MF. Nonessential research in the new normal: the impact of COVID-19. Am J Trop Med Hyg. 2020;102:1164–5. 32333545 10.4269/ajtmh.20-0325 PMC7253117

[4] Queensland Health. Revoked and superseded public health directions. State of Queensland. https://www.health.qld.gov.au/system-governance/legislation/cho-public-health-directions-under-expanded-public-health-act-powers/revoked. Accessed February 16, 2025.

[5] Australian Bureau of Statistics. COVID-19 mortality in Australia: deaths registered until 31 January 2023. Australian Bureau of Statistics. https://www.abs.gov.au/articles/covid-19-mortality-australia-deaths-registered-until-31-january-2023#cite-window1. Accessed February 16, 2025.

[6] Johns Hopkins University Coronavirus Resource Center. Mortality analyses. Johns Hopkins University and Medicine. https://coronavirus.jhu.edu/data/mortality. Accessed February 16, 2025.

[7] Klomjit N Zand L Cornell LD Alexander MP. COVID-19 and glomerular diseases. Kidney Int Rep. 2023;8:1137–50. 37274308 10.1016/j.ekir.2023.03.016 PMC10041821

[8] Australian Government. Australian Health Protection Principal Committee (AHPPC) statement on the public health implications of the Omicron variant and response options. Department of Health, Disability and Ageing. Updated 22 December, 2021. https://www.health.gov.au/news/ahppc-statement-on-the-omicron-public-health-implications-and-response-options. Accessed September 15, 2025.

[9] Ellis RJ Moffatt CR Aaron LT. Factors associated with hospitalisations and deaths of residential aged care residents with COVID-19 during the Omicron (BA.1) wave in Queensland. Med J Aust. 2023;218:174–9. 36524321 10.5694/mja2.51813 PMC9877866

[10] Bromme R Mede NG Thomm E Kremer B Ziegler R. An anchor in troubled times: trust in science before and within the COVID-19 pandemic. PLoS One. 2022;17:e0262823. 35139103 10.1371/journal.pone.0262823 PMC8827432

[11] Bhutkar R El-Den S O’Reilly CL Collins JC. The impact of COVID-19 on clinical research at Australian and New Zealand universities: a qualitative study. Collegian. 2023;30:612–9. 10.1016/j.colegn.2023.05.002 PMC10165013 37360918

[12] Peeters A Mullins G Becker D Orellana L Livingston P. COVID-19’s impact on Australia’s health research workforce. Lancet. 2020;396:461. 10.1016/S0140-6736(20)31533-6 PMC7426082 32798480

[13] Park JJH Mogg R Smith GE. How COVID-19 has fundamentally changed clinical research in global health. Lancet Glob Health. 2021;9:e711–20. 33865476 10.1016/S2214-109X(20)30542-8 PMC8049590

[14] Glasziou PP Sanders S Hoffmann T. Waste in covid-19 research. Bmj. 2020;369:m1847. 32398241 10.1136/bmj.m1847

[15] May RM Cassol C Hannoudi A. A multi-center retrospective cohort study defines the spectrum of kidney pathology in Coronavirus 2019 Disease (COVID-19). Kidney Int. 2021;100:1303–15. 34352311 10.1016/j.kint.2021.07.015 PMC8328528

[16] Kudose S Batal I Santoriello D. Kidney biopsy findings in patients with COVID-19. J Am Soc Nephrol. 2020;31:1959–68. 32680910 10.1681/ASN.2020060802 PMC7461665

[17] de Las Mercedes Noriega M Husain-Syed F Wulf S. Kidney biopsy findings in patients with SARS-CoV-2 infection or after COVID-19 vaccination. Clin J Am Soc Nephrol. 2023;18:613–25. 36723286 10.2215/CJN.0000000000000106 PMC10278827

[18] Emanuel EJ Persad G Upshur R. Fair allocation of scarce medical resources in the time of covid-19. N Engl J Med. 2020;382:2049–55. 32202722 10.1056/NEJMsb2005114

[19] Burke JP Pham T May S. Kidney biopsy practice amongst Australasian nephrologists. BMC Nephrol. 2021;22:291. 34445981 10.1186/s12882-021-02505-9 PMC8390249

